# Evaluation of a Spiritual History with Elderly Multi-Morbid Patients in General Practice—A Mixed-Methods Study within the Project HoPES3

**DOI:** 10.3390/ijerph19010538

**Published:** 2022-01-04

**Authors:** Ruth Mächler, Noemi Sturm, Eckhard Frick, Friederike Schalhorn, Regina Stolz, Jan Valentini, Johannes Krisam, Cornelia Straßner

**Affiliations:** 1 Department of Psychosomatic Medicine and Psychotherapy, Professorship for Spiritual Care and Psychosomatic Health, Technical University of Munich, Langerstr. 3, 81675 Munich, Germany; eckhard.frick@tum.de; 2 Department of General Practice and Health Services Research, University Hospital Heidelberg, Im Neuenheimer Feld 130.3, 69120 Heidelberg, Germany; noemi.sturm@med.uni-heidelberg.de (N.S.); cornelia.strassner@med.uni-heidelberg.de (C.S.); 3 Institute of General Practice and Interprofessional Care, University Hospital Tübingen, Osianderstr. 5, 72076 Tübingen, Germany; Friederike.Schalhorn@med.uni-tuebingen.de (F.S.); regina.stolz@med.uni-tuebingen.de (R.S.); jan.valentini@med.uni-tuebingen.de (J.V.); 4 Institute of Medical Biometry, University Hospital Heidelberg, Im Neuenheimer Feld 130.3, 69120 Heidelberg, Germany; krisam@imbi.uni-heidelberg.de

**Keywords:** spirituality, health, holistic care, family doctor, spiritual history, spiritual needs assessment, religious coping, elderly patients, doctor-patient relationship, general practitioner, primary care

## Abstract

Background: The “Holistic Care Program for Elderly Patients to Integrate Spiritual Needs, Social Activity and Self-Care into Disease Management in Primary Care” (HoPES3) examines the implementation of a spiritual history (SH) as part of a multifaceted intervention in German general practices. While the effectiveness of the interventions was evaluated in a cluster-randomized trial, this article investigates the patients’ views concerning the acceptability of the SH and its effects. Methods: A mixed-methods study was conducted in which 133 patients of the intervention group filled in a standardized questionnaire after the intervention. Later, 29 of these patients took part in qualitative semi-standardized interviews. Results: According to the survey, 63% (*n* = 77) of patients found the SH helpful. In the interviews, however, many indicated that they either kept the conversation brief or declined the offer to talk about spirituality. Contents of longer conversations referred to difficult life events, personal sources of strength, and experiences with religious institutions. Many patients who had a longer conversation about spirituality reported that their relationship with their general practitioner (GP) had improved. Almost all patients recommended integrating a personal conversation of this kind into primary care. Conclusions: The SH seems to be a possible ‘door opener’ for a trusting doctor-patient relationship, which can then be built upon.

## 1. Introduction

Due to the demographic change and improved treatment options, the proportion of elderly patients suffering from multiple chronic conditions and treated with long-term medication is constantly increasing in industrial countries. It is well known that this patient group experiences a significantly lower quality of life. Besides the disease burden, social and psychological factors contribute to this. For example, multimorbidity is often associated with loneliness, hopelessness, and the feeling of powerlessness [1,2,3].

The idea that the spiritual dimension is relevant for health is finding increasing acceptance in health care. In the context of this study, we defined spirituality as anything that is meaningful to the patient’s life and serves as a personal resource. The definitions of spirituality and spiritual needs are very heterogeneous but often have four attributes in common: Connectedness (e.g., to feel connected to the world or other people, to talk to someone about personal issues), transcendence (e.g., to immerse oneself in nature, to pray), peace (e.g., to find inner peace, to relax in a peaceful place), and meaning in life (e.g., to pass on life experience, to be sure that one’s own life is purposeful) [4,5]. A broader definition of spirituality was chosen for this study to emphasize that spirituality encompasses more than religion.

In the context of care, it is important to understand that spirituality is considered a dimension of being human, meaning that a person is not either spiritual or not, but that all humans have spiritual needs, which however differ in form and content.

Thus, medical facilities, medical practices, hospitals, nursing homes, facilities for the disabled, or rehabilitation clinics should explore patients’ spirituality and provide support in this area. It is generally assumed that GPs are well suited to integrate older adults’ living circumstances and biographical influences into their health care [6,7,8,9]. This is because they are usually the first contact person for all health issues and often accompany their patients for years. GPs also increasingly recognize the importance of the spiritual dimension in primary care [6,10,11,12,13,14,15,16].

This is not only the GPs’ view. Patients also wish for their doctors to take their spiritual needs into account. A literature analysis of numerous studies by Best et al. has revealed that, in fact, many patients would like to talk about their spiritual issues with those involved in their medical care [17]. The same applies to German patients as reported by hospitalized cancer patients [18] and outpatient pain patients [19].

Spirituality has the potential to be a major resource, especially for older adults as they age and have to cope with the hardships and challenges that inevitably occur in later life [20,21,22,23]. A representative survey among Germans revealed that the individual factors determining the meaning of life change with age. While younger people mention “friends”, “partnership”, or “work” as decisive factors, spiritual issues are most important to patients aged 70 or older. In a study conducted in German nursing homes, “praying for oneself”, “reflecting on previous life (events)”, and “participating in a religious ceremony” were the highest scored needs, followed by giving needs (giving solace to someone, passing on one’s life experience) and inner peace needs (plunging into nature, turning to someone in a loving attitude) [24].

Although both patients and GPs find it useful to talk about spiritual issues, it is unclear how often this actually happens. Studies from different countries show that there is a great deal of uncertainty on the part of GPs in terms of both terminology and time commitment [5,12,22,23]. Since in German-speaking countries spiritual care is less established than in English-speaking health care, it is likely that German GPs face similar or even more serious problems [25,26].

In Germany, GPs’ efforts to improve older patients’ health and quality of life usually focus on standardized programs, such as the disease management programs (DMP). DMPs are based on regular appointments every 3–6 months, during which a standardized diagnostic and treatment protocol has to be completed (for example monitoring defined blood parameters, physical examinations, review, verification of vaccination status, and structured referrals). The importance of such measures is undeniable. However, there is a risk of neglecting other dimensions of care which are less obvious and more difficult to measure, specifically spiritual needs, self-care, and social inclusion.

The “Holistic Care Program for Elderly Patients to Integrate Spiritual Needs, Social Activity and Self-Care into Disease Management in Primary Care” (HoPES3) examines the implementation of an SH as part of a multifaceted intervention in German general practices [27].

As explained in the ‘Study Design’ below, the present study was part of the process evaluation of the cluster-randomized controlled HoPES3 trial [27,28] and focusses on the acceptability, feasibility, benefits, and harms of the SH from the perspective of the participating patients. This article intends to answer four specific research questions:

1. To what extent do patients accept the offer to get involved in an SH?
2. What do patients talk about when invited to speak about their spirituality?
3. What are the positive and negative effects of the SH from the patients’ point of view?
4. What do patients recommend to their doctors after having taken part in the SH?

## 2. Materials and Methods

The reporting of methods and results follows the Consolidated Criteria for Reporting Qualitative Research (COREQ) [29].

### 2.1. Study Design

This study was part of the HoPES3 project [27] funded by the German Ministry of Education and Research (funding code 01GL1803) and registered in the German Clinical Trials Register (DRKS00015696). Ethics approval was obtained from the Ethics Committee of the University of Heidelberg (reference number S-730/2018). Within the scope of the HoPES3 project, a complex intervention (described below) to strengthen the consideration of patients’ spiritual needs, social activities, and self-care abilities in primary care was developed and evaluated in a cluster-randomized control trial with accompanying process evaluation. While the statistical analyses showed, along with other effects, a notable improvement of mental well-being in patients who stated that spirituality was important to them, this mixed-methods study was part of the process evaluation and aimed to assess patients’ experiences with the SH, which was one component of the complex intervention. For this purpose, the patients of the HoPES3 intervention group were invited to participate in a semi-structured telephone interview (conducted in the period from February to June 2020) and a written survey (completed within two weeks after the SH).

### 2.2. Reflexivity

First author Ruth Mächler (Dr. rer. pol.) reflected on her own role, possible bias, and influence on the research as well as her first choice of codes at the beginning of the coding phase within the framework of a Qualitative Workshop, conducted at the Ludwig-Maximilians-Universität München. The analyses, the results, and the first draft of the manuscript were discussed with the research team.

### 2.3. Context and Theoretical Framework

A theoretical model specifying the assumed effect mechanism of the HoPES3 intervention was developed before the intervention started, and published in the study protocol [27]. The aim of HoPES3 was to increase older patients’ self-efficacy and quality of life by raising awareness of their spirituality and spiritual needs and by promoting social contacts and self-care. The latter included activities patients can do on their own with the exception of taking medication to increase their well-being. Inclusion criteria for patients were: Aged 70 years or older, suffering from at least three chronic diseases, taking at least three medications, participating in at least one DMP, and being able to participate actively in the study.

The HoPES3 intervention comprised several components. GPs were trained in a 5-h workshop to assess a spiritual history according to the instrument SPIR, the German-language adaptation of the US-American interview guide FICA [30]. This conversation guide allows GPs to explore patients’ spiritual and religious orientation using four open-ended questions that encourage conversation about any existential issue or personal need [15,29,30]:

**S**: Would you describe yourself in the broadest sense of the term as a believing/spiritual/religious person?

**P**: What is the place of spirituality in your life? How important is it in the context of your illness?

**I**: Are you integrated in a spiritual community?

**R**: What role would you like to assign to your doctor in the domain of spirituality?

The questions are supplemented by sub-questions, which can complement or replace the questions (see Appendix A).

The SH was supposed to raise patients’ awareness for their own spirituality and sources of strength. It was also assumed that the GP’s offer to talk about the patient’s spirituality as well as the potential conversation would have an influence on the doctor-patient relationship.

In addition, medical assistants provided information about social activities for seniors in the region and/or about non-pharmacological interventions (exercises, home remedies) to reduce frequent complaints in old age. Patients in the intervention group were supposed to receive this offer at their regular DMP appointment, whereas DMP appointments in the control group were carried out as usual.

### 2.4. Recruitment of the Trial Participants

To recruit GP practices for the trial, all family practices in defined areas around Heidelberg and Tübingen were contacted by mail. To recruit patients, GPs invited all patients meeting the inclusion criteria (see above) and having a DMP appointment within the next three months to participate in the study. Finally, 14 general practices and 177 of their patients were randomized to the intervention group and 14 general practices and 146 of their patients were randomized to the control group. After 4 practices had withdrawn in the course of the study, data from 24 practices and 297 patients completed the trial

### 2.5. Data Collection for the Process Evaluation

The patients of the intervention group were asked to fill in a questionnaire within two weeks of the spiritual history. One hundred and thirty-three completed questionnaires were returned. Questionnaires were digitized and validated by three members of the study team (CH, MB, and NSt) and analyzed descriptively using IBM SPSS Statistics 25 (IBM Corporation, Armonk, NY 10504-1785, USA).

All patients of the intervention group were invited to participate in a semi-structured telephone interview (Interview Guide see Appendix B). All those who declared their interest in participation by completing a return form were contacted by phone. Interviews were carried out after the SH from February until June 2020. In total, 29 qualitative interviews were conducted. During the first two months, the respondents could choose between face-to-face and telephone interviews. Later, due to the COVID-19 pandemic, only telephone interviews were possible. Hence, some of the interviews took place at the patients’ homes, while others were conducted by phone. The duration of the interviews varied between 14 and 42 min. Each interview was conducted only once, no field notes were taken, and transcripts were not returned to participants.

### 2.6. Analysis

All qualitative data was included for analysis. (While questions were also asked about the other intervention components, only the material related to the SH is reported here). Audio recordings were pseudonymized and transcribed verbatim by a research assistant and RM following predefined transcription rules. The MAXQDA Analytics Pro 2020 (Release 20.0.8) software (VERBI SOFTWARE-CONSULT-Sozialforschung, 35287 Amöneburg, Germany) was used for data management, coding, and content analysis.

The analysis was performed with Reflexive Thematic Analysis [31,32,33]. First, main categories were identified deductively based on the interview guideline and inductively from the material. Next, sub-categories were added inductively. Themes were reviewed by identifying the ‘essence’ of each theme. Subsequently, the relationship between the themes was examined, as well as their place in the overall story.

In this article, only the results referring to the SH are reported.

## 3. Results

### 3.1. Characteristics of the Sample

Table 1 shows the sociodemographic characteristics of the participants. Of the 146 patients randomized to the intervention group, 133 completed the survey and 29 participated in an interview. The characteristics of participants who only completed the survey but were not interviewed, and those who took part in an interview, were largely comparable. Considerable differences were only detected regarding the educational level. While 21% (*n* = 6) of the interviewees had a university degree, this corresponded to only 8% (*n* = 8) of the survey participants.

### 3.2. Results of Survey and Interviews

In the following, each research question will be answered combining the findings from the survey and the interviews for the purpose of triangulation.

*To what extent do patients accept the offer to conduct a spiritual history?*

From among the 29 interviewees, 38% (*n* = 11) stated that they really engaged in the conversation about their spirituality. The remainder of patients (62%, *n* = 18) declined the offer to talk about spirituality, except for two patients who explicitly reported not having been invited to talk about their own spirituality.

The survey shows a different ratio of accepted and declined SHs (see Table 2, Item 1), which will be discussed later. Two-thirds of the SHs lasted 20 min or less (see Table 2, Item 2).

*What do patients talk about when invited to speak about their spirituality?*

According to the written survey, the conversation lasted 20 min or less in the majority of cases (62%, *n* = 82), and in 19% (*n* = 25) between 21 and 30 min (Table 2, Item 2). NB: It is possible that some patients were referring not only to the SH, but to the entire appointment, including the consultation with the medical assistants.

According to the interviewees, the content of the longer conversations between the patients and their doctors repeatedly concerned serious life events in the past: Illness or death of the participants’ children, siblings, or life partners; broken marriages; and broken relationships with close relatives. Another focus of the SH was to look at patients’ personal sources of strength. In this context, relationships with family members and life partners were often mentioned. Friendships and good neighborly relations were also considered. Furthermore, activities and hobbies—such as handicrafts, making music, traveling, reading and sports—were brought up. Nature was frequently mentioned as helping to clear one’s mind or to reenergize, or it was described as a place to find hope. This was exemplified by walks in the countryside, hikes in the Swabian Alps, and gardening. Other respondents stated religion and spiritual experiences to be important sources of strength and hope.

The subject of religion and belief was ambivalent. For some respondents, their religious beliefs and experiences were very important sources of strength and hope. (For quotes in original language see Appendix C.)

PAT: Then she asked me what I drew my strength from. She also knows a bit about something like that from my life. So right now, with the family, with my children, whenever there is a problem, I can talk to her about it. And then I also told her about my faith, I am a New Apostolic, and that I draw a lot of strength from there and that I can also turn to God with confidence when things get really tight and difficult.

Some reported having difficulties with the church. This was also given as a reason for rejecting the offer to talk about spirituality in the first place. Others described the social and community functions of the church as a source of strength which allowed them to get involved in voluntary work, for example.

Overall, personal stories were shared more often during longer SHs. The patients took the SH as an opportunity to tell the GP their personal story and to give them an insight into their life and thoughts.

PAT: Well, she got everything out of me in terms of my life, what ambitions I have, how I (.) things like religiosity and environment and neighbors. Well, so she looked at everything that was going on in my life.

INT: And how did you feel about the conversation?

PAT: Very good.

*What are the positive and negative effects of the spiritual history from the patients’ point of view?*

In the survey, more than half of the respondents (63%, *n* = 77) stated that the conversation was quite or very helpful, about one third (37%, *n* = 45) stated that it was of little or no help (see Table 2, Item 3). Hardly anyone felt that the conversation was somewhat or very stressful (Table 2, Item 4)

None of the 29 interviewees reported any negative emotions as a result of the SH, nor did they wish they had not had the SH. How the GPs conducted the conversation was not criticized at any point, even if the setting was described as unfavorable in some cases, for example because the waiting room was full or the questions during the usual appointment came as a surprise for the patients.

None of the patients reported having changed anything later in life or having tried new things as a result of the SH, but many patients reported that as a result of the SH their relationship with their GP had improved and deepened. Eleven patients reported having had a longer conversation about spirituality with their GP. Of those, 7 reported an improved relationship and 4 either already had a good relationship or reported no change. None of them said that the relationship had worsened in any way. Four others, who said they had talked only briefly about spirituality but for longer about social activities, also reported an improved doctor-patient relationship.

PAT: Well, I just think through the conversation you get to know each other better or she’ll know a little more about me, like my life’s journey, and well, I think that is very positive for further treatments, so, I found that very good.

INT: Does it continue to have an effect?

PAT: Er, yes. In this respect, because then you (...) that increases your trust in the doctor.

In addition, patients expected that they would be able to talk about personal issues more easily in the future.

PAT: I was comfortable with that. Well, because then, it is so open then, this barrier is broken if you can talk to the doctor about everything.

PAT: Well, I don’t see any more inhibitions there. (…)

PAT: I can come in and can, can simply say when help is urgently needed.

As a result of the SH some patients reported that they now feel less stressed whenever they need to see their GP.

PAT: But now I don’t feel the same pressure anymore like I used to. When I had to see the doctor my blood pressure was already very high. And I don’t have that anymore. (.) In relation to this. If I’ve got something I need to see her for, (.) I don’t have this outburst anymore, right? 160, 180 blood pressure, just by walking through the door.

*What do patients recommend to their doctors after participation in the spiritual history?*

As part of the interviews, patients were asked whether they would recommend implementing an SH into general practice. Almost all patients recommended the offer of such a conversation, although some of them also suggested restrictions:

Patients assumed that in general, GPs are not willing or able to take the time for such conversations.

PAT: Well, but you know (.) doctors don’t have the time. That you have very long conversations (2 s) that, well, especially to get to know someone, it is certainly not unimportant, but (3 s), well, I don’t think the time is there, on the doctor’s side.

The respondents repeatedly mentioned that it is of great importance that GPs emphasize the voluntary character of the SH and do not put any pressure on patients.

PAT: Yes, everything voluntarily.

INT: Voluntariness is important to you?

PAT: Yes, exactly.

INT: And based on this experience, do you have any recommendations for your doctor? If he should, should he even address such things or, if so, how? Do you have anything where you say ...

PAT: That actually depends on the patient, whether they are willing to respond. So it will differ from case to case, I really don’t want to give any judgment, let’s put it that way. He addressed it, I accepted the offer. With the next patient …that is up to each individual patient.

In the context of the study, SHs were integrated into patients’ DMP appointments which took place at a set date at a predetermined moment. Even though the patients were aware of taking part in the HoPES3 study, many of them were surprised by the questions posed by their GPs. Some recommended afterwards that GPs should raise such issues only if there is a need and at an appropriate moment.

INT: Would you have a recommendation for doctors based on your experiences now with these conversations?

PAT: They should start listening attentively, if the patients somehow (-)er, bring up this kind of thing.

INT: A doctor should pay attention to that and then respond to it?

PAT: Yes, pay attention to that, exactly. And if, maybe, I don’t know how this will be, whether problems will come up or something like that, or if he feels very bad, something like that, then go into it.

Another restriction that the patients mentioned referred to the individual suitability of GPs. Some respondents expressed doubts about whether all GPs had the ability to hold spiritual conversations.

Many patients also stated that they themselves would not have needed the interview but that many other people certainly benefited from such conversations. Therefore, in most of the patients’ views, GPs should generally make the offer to talk about spirituality.

## 4. Discussion

This mixed-methods study examined patient perspectives on the acceptability and effects of an SH in general practice. Some of the main findings will be discussed below in relation to the four research questions with reference to the international literature.

*To what extent do patients accept the offer to conduct a spiritual history?*

In our study, quantitative and qualitative data seemed to differ with respect to the acceptance of the SH. Only 38% (11 out of 29) of patients interviewed stated that they had had an SH in the sense that they opened up during the conversation. In the written survey, however, 92% of the patients answered in the affirmative the question whether they had had a conversation about their personal sources of strength and / or questions of faith with their GP. A detailed comparison showed that the data in the questionnaire and in the interview did indeed differ at the individual level. It could be that patients responded to the offer to talk about spirituality either very briefly or not at all, but nevertheless rated this as a conversation in the questionnaire. In the semi-standardized interviews, however, they then frequently explained that they had not become further involved. This supports the above-mentioned hypothesis that the offer of an SH is already perceived as an intervention (and may have long-term intervention effects) even if it does not result in a longer conversation.

Another explanation would be that the patients had only rejected the SH itself but had, in fact, talked with the GP about other personal issues. A third explanation for these divergent results could be a selection bias among the interviewees in favor of patients who had a predominantly negative attitude towards the SH. Yet the data provided no such hints.

The frequent rejection of talking about spirituality may have several causes, one of which could be that patients’ own perspectives on spirituality differ widely. Further reasons are discussed in depth in another paper [34].

*What do patients talk about when invited to speak about their spirituality?*

The SH lasted 20 min or less in the majority of cases and a further 19% remained below 30 min. This is not particularly long, considering that GPs’ concerns about not being able to devote the time to long conversations is one of the main barriers, according to the literature on spiritual care.

Research shows that spirituality can be a resource to promote and maintain resilience in later life and to overcome hardships [20]. However, in our study many participants reacted with reluctance to questions about their spirituality mainly because they confused this term with institutionalized religion. Our data suggests that patients are far more open to talking about decisive life events and personal sources of strength than about their religion or faith in God. Therefore, GPs should choose appropriate wording when introducing the SH and only use prejudiced terms, such as church, religion, belief, or spirituality at the beginning of the SH if they are sure that patients understand them in the right way. On the other hand, for some patients, religious beliefs may be an important part of their spirituality. Therefore, they should not be avoided or made taboo in the conversation. It seems to be reasonable to introduce those topics later on in the SH, carefully avoiding pressure or the impression that judgment is being passed, but making sure not to omit them. A study by Vermandere found that “communicating willingness to engage in spiritual care, using a non-judgmental approach, facilitates spiritual conversations” [35]. During the training within the HoPES3 study this strategy was already recommended to the doctors participating, emphasizing that the SPIR instrument should serve as guidance but is not meant to offer preformulated questions. Looking at the participants’ experiences, we conclude that these recommendations need to be addressed more clearly and with more emphasis in order to be effectively implemented and to avoid any confusion between spirituality and institutionalized religion.

It was striking how important it was for the patients to talk about their biography, especially about serious life events in the past. Patients seemed to be primarily concerned with explaining their current situation, outlook on life, and spirituality to the primary care physician based on their life history. The importance of reflecting on one’s own biography in old age—also for maintaining good health is for example emphasized in Erikson’s concept of “ego integrity“ which describes eight stages of psychosocial development. When a person reaches the last of these stages—integrity versus despair—the person will look back and reflect on the life they have led and look into the future, knowing that death is near. Thus, they face the task of accepting their biography and the person they have become. Accomplishing this allows the person to experience “integrity”, to make peace with themselves, and to find meaning even in the face of the inevitability of death. Those who do not achieve integrity are found to succumb to despair, regret not having achieved their life goals, suffer from the injustice of life, and dread death [36,37,38]. Taking a spiritual history in the primary care setting supports older patients in this important psychological process. Therefore, they responded positively to the GP’s/physician’s interest in them and their story.

*What are positive and negative effects of the spiritual history from the patients’ point of view?*

The main positive effect of an SH reported by patients who had experienced an in-depth SH was an improved relationship with their GP, particularly with regard to increased trust, better understanding, and less tension when visiting the doctor. Similar findings are described in other studies, for older as well as for younger patients. For example, an intervention study with 40 schizophrenic patients demonstrated improvements concerning treatment satisfaction and patient-physician-relationships after the SH [39]. Another study showed that the instrument was found helpful by both physicians and patients in addressing spiritual issues, planning further treatment, and strengthening the physician–patient relationship [40]. From psychotherapy research, the importance of the “therapeutic alliance” between a patient and a doctor or therapist is well known [41,42,43,44]. The therapeutic alliance consists of three essential elements: Agreement on the goals of the treatment, agreement on the tasks, and the development of a personal bond made up of reciprocal positive feelings. This bond is strengthened by the conversation on spirituality between the GP and the patient.

Only 2% of patients experienced the SH as quite or very stressful and no other negative effects were reported by the participating patients. This is consistent with the findings of a study by Frick et al. evaluating the instrument SPIR [18]. Interestingly, patients did not experience stress even if they had declined the offer to talk about spiritual issues. Therefore, GPs can be encouraged to offer an SH proactively and not to avoid it for fear of patients’ reactions. Even if the offer is rejected, it signals to patients that their GPs are open to discussing such topics should they wish to do so at a later time. This was also confirmed by the interviewees who stated that after the SH it would be easier for them to raise personal issues during meetings with their GP. Therefore, we assume that this “message to patients” will have an impact in the long run but since interviews were conducted shortly after the offer of the SH, later developments could not be recorded. Further research should focus on the long-term effects of an SH on outcomes such as patient–physician relationship, course of treatment, and on health and quality of life, especially in patients who had initially turned down participation in the conversation.

*What do patients recommend to their doctors after participation in the spiritual history?*

Almost all patients stated that they would appreciate the implementation of SHs at their GP’s practice, although some expressed concerns regarding GPs’ lack of time to adequately respond to patients’ needs and also concerns around the attitude/ability of the doctor to carry out SHs. It would be recommended that GPs receive training in spiritual care [45]. Another important concern of patients was that the process was voluntary. Since declining the offer of the SH was not perceived as stressful by the participating patients, it can be concluded that the GPs clearly communicated the voluntary nature of the SH. Protection of the patient’s autonomy is generally recognized as an important condition for a successful SH [18].

*Proactive or reactive approach and timing.*

The timing of the invitation to talk also matters. In the study, timing for the SH was set artificially, whereas normally the GP could decide when to address the topic. This can be right at the start of a consultation as part of the general history or if it is obvious that the patient has a need or when the patients bring something up themselves. The last is an alternative that is preferred in the literature by some, while others recommend the proactive approach, as is the case with some of the patients in our study (for the discussion about this see: [45,46,47,48,49]. The advantage of proactively addressing the topic is that the benefits of an SH will reach more patients. A reactive approach could result in missing out patients who do not appear to have a definite need for an SH, but who would actually benefit from it. GPs can identify those patients for example by routinely including an assessment of individual beliefs and resources in the medical history of chronically-ill patients.

The fact that many respondents spoke very openly about their spirituality during the qualitative interview, in which the interviewer was completely unknown to them, suggests that an open conversation is possible at an early stage of the doctor–patient relationship, so that conducting an SH could already be offered during one of the first consultations.

One of the essential questions of the HoPES3 study was whether an SH can be a helpful instrument in general practices [11,27,50]. According to this process evaluation, this can be affirmed, if the intention of the SH and the correct use of the instrument is well understood.

## 5. Strengths and Limitations

To our knowledge, this is the first study assessing the experiences of German GPs with an SH. The combination of qualitative and quantitative data allowed triangulation and thus a better understanding of the findings. The findings of this study have to be interpreted with regards to some limitations: The study sample is probably not representative of patients of German GP practices as a whole. The GPs-who themselves probably tended to have special interest in holistic health care-recruited the patients. This may have resulted in a bias towards patients with better communication skills and better health. Interviews were performed between a couple of weeks up to two months after the intervention. Therefore, it cannot be ruled out that participants did not remember every detail about the conversations with their GPs. During the first two months, the respondents could choose between face-to-face and telephone interviews. Later, due to the COVID-19 pandemic, only telephone interviews were possible. This could have influenced the results.

## 6. Conclusions

Conducting an SH is likely to foster a trusting doctor–patient relationship, which can be built upon and used in later crisis situations. Since the SH was a valuable experience for those patients who followed the invitation and not stressful for those who declined it, the implementation of an SH in primary care may should be considered and further investigated. When training doctors in conducting SHs, patients’ concerns should be incorporated.

## Figures and Tables

**Table 1:** Sociodemographic data of the sample.

	Written Survey Only	Interview	Total	*p*-Value (Written Survey Only vs. Interview)
Number of patients	107 (100%)	29 (100%)	136 (100%)	-
Participated in written survey	107 (100%)	26 (89.7%)	133 (97.8%)	-
Mean age in years (range, SD)	78.37 (70–91, 4.59)	76.83 (70–85, 4.68)	78.04 (70–91, 4.64)	0.120 ^a^
Female *n* (%)	61 (57.0%)	13 (44.8%)	74 (54.4%)	0.243 ^b^
Living alone *n* (%)	36 (35.6%) 6 missings	7 (24.1%) 3 missings	43 (32.3%) 9 missings	0.402 ^b^
Highest level of education *n* (%)				0.055 ^b^
Primary and secondary school education	89 (88.1%)	18 (62.1%)	104 (78.1%)	
High school (German final school exams)	4 (4.0%)	2 (6.9%)	6 (4.7%)	
University degree	8 (7.9%)	6 (20.7%)	14 (11.0%)	
Missings	6 missings	3	3 missings	
Mean number of medications (range, SD)	7.67 (3–17, 3.43)	8.00 (3–20, 3.68)	7.74 (3–20, 3.47)	0.669 ^a^
Religion *n* (%)				0.877 ^b^
Christian	89 (88.1%)	23 (85.2%)	112 (87.5%)	
Other	5 (5.0%)	2 (7.4%)	7 (5.5%)	
No religion	7 (6.9%)	2 (7.4%)	9 (7.0%)	
Missings	6 missings	2 missings	8 missings	

^a^: unpaired *t*-test, ^b^: chi-squared-test.

**Table 2:** Results of the written survey (*n* = 133).

	% (*n*)
1. Have you had a conversation with your GP in the last 2 weeks about your personal sources of strength and/or faith issues?	
No	7.5 (10)
Yes	92.5 (123)
Missing	0.0 (0)
2. How long did this conversation last?	
1–10 min	17.3 (23)
11–20 min	44.4 (59)
21–30 min	18.8 (25)
>30 min	12.0 (16)
Missing	7.5 (10)
3. How helpful did you find the conversation?	
Not at all	2.3 (3)
A Little	31.6 (42)
Quite	33.8 (45)
Very	24.1 (32)
Missing	8.3 (11)
4. How stressful did you find the conversation?	
Not at all	73.7 (98)
A Little	15.8 (21)
Quite	1.5 (2)
Very	0.8 (1)
Missing	8.3 (11)

## Data Availability

Selected data may be made available upon reasonable request.

## Appendix A. SPIR Guideline

The SPIR acronym is used to visualize the four steps in conducting a spiritual history

**S** piritual and faith beliefs

**P** lace and influence of these beliefs in patient’s life

**I** ntegration into a spiritual, religious, ecclesiastical community/group

**R** ole of the physician: How should the physician deal with spiritual expectations and patients´ problems?

The following standard questions should be adapted to the patient’s use of language during the SH. To avoid misunderstandings, it should be found out whether patient is familiar with terms such as “spiritual” or “religious” and how it is used by patient. The same applies to church/community/congregation/group, etc., depending on how the patient is able to talk about his or her ties in this regard.

**S**: Would you describe yourself in the broadest sense of the term as a believing/spiritual/religious person?

In whom or in what do you place your hope?

From what do you draw strength?

Is there something that gives your life meaning? What beliefs are important to you?

**P**: What is the place of spirituality in your life? How important is it in the context of your illness?

How have your spiritual and faith beliefs determined your behavior during this illness?

What role do your beliefs play in helping you get well?

**I**: Are you integrated in a spiritual community?

To what extent does this mean support for you?

Is there a person or group of people who really mean a lot to you and who are important to you? Would you wish to have more companionship?

**R**: What role would you like to assign to your doctor in the domain of spirituality?

Who is your most important dialog partner in terms of spiritual and faith beliefs?

What role should these beliefs play in medical treatment?

Spiritual and faith issues are an important area in sickness and in health. Do you feel that we have talked about your beliefs in the way you would like?

Is there anything you would like to add?

## Appendix B. Interview Guide (Process Evaluation Spiritual History Only)

- Have you had a conversation with your primary care physician in the last few months about spirituality or about what gives you personal strength and meaning in life?
- If no: Why not?
- If yes: How was this conversation for you and how did you feel about it?
- Please tell me how you felt after the conversation
- Did you do anything new or different afterward?
- Did the conversation have any further impact or aftermath for you?
- Did the conversation have an impact on your relationship with your primary care physician?
- What would you recommend to physicians based on your experience with this conversation?
- What is spirituality for you?
- How would you describe your personal spirituality?
- What do you take away from the study for the future?
- Would you like to see all primary care practices offer a program like the HoPES3 study for older patients?

## Appendix C. Quotes in Original Language

*PAT: Sie hat dann auch mich gefragt, woraus ich so Kraft schöpfe. Sie kennt auch ein bisschen, so in etwa schon mal so etwas aus meinem Leben. Also jetzt gerade von der Familie, von Kindern, wo es da immer mal ein Problem gibt, wo ich auch mit ihr drüber reden kann. Und ich habe ihr dann auch gesagt, dass ich eigentlich aus meinem Glauben, ich bin neuapostolisch, dass ich da also sehr viel Kraft schöpfe und mich auch vertrauensvoll dann an den lieben Gott wenden kann, wenn es mal ganz eng und schwer wird.*

*PAT: Ja sie hat alles aus, aus mir rausgeholt was mein Leben betrifft, welche Ambitionen ich so habe, wie ich, (.) so etwas Religiöses und Umfeld und Nachbarn. Und sie hat also alles so sich angeguckt, was bei mir los ist.*

*INT: Und wie ging es Ihnen da so mit dem Gespräch?*

*PAT: Sehr gut.*

*PAT: Ja, ich denke einfach man lernt sich durch das Gespräch noch besser kennen oder sie weiß dann etwas mehr von mir, so meinen Lebensweg, und ja. Ich denke, das ist sehr positiv auch für weitere Gespräche oder Behandlungen, also ja, das fand ich jetzt sehr gut.*

*INT: Hat da vielleicht noch etwas nachgewirkt?*

*PAT: Ähh, ja. Insofern, weil man dann ein (…) dass man größeres Vertrauen zum Arzt bekommt.*

*PAT: Mir war das angenehm. Also eben, weil es dann so offen, da, da ist dann diese Barriere aufgebrochen, wenn man mit dem Doktor auch über alles reden kann.*

*PAT: Also, ich sehe da jetzt keine Hemmschwelle mehr drin.*

*INT: Keine Hemmschwelle mehr. Mhm, genau.*

*PAT: Ich kann da rein und kann, kann einfach sagen, wo es brennt.*

*PAT: Aber ich habe jetzt nicht mehr den Druck so wie vorher, wenn ich zum Doktor musste war mein Blutdruck ja schon ganz oben.*

*INT: Ja.*

*PAT: Und das habe ich jetzt nicht mehr. (.) Also was sich auf dieses bezieht. Wenn ich irgendetwas habe, wo ich zu ihr rein muss, (.) da habe ich nicht mehr diesen Ausbruch, ne? 160, 180 Blutdruck, bloß, weil ich da zur Tür reingehe.*

*PAT: Ja, aber wissen Sie, (.) die Zeit haben die Ärzte ja nicht. Dass, dass man da so ewig lange Gespräche führt, die, ja, gerade zum Kennenlernen des Menschen ist sicherlich nicht unwichtig, aber (3 s), ja, ich glaube nicht, dass die Zeit da ist, vom Arzt her.*

*PAT: Anbieten, ja*

*INT:.Mhm*

*PAT: Ja, alles freiwillig*

*INT: Ja, Freiwilligkeit ist Ihnen wichtig?*

*PAT: Ja, genau*

*INT: Und aufgrund dieser Erfahrung, hätten Sie irgendeine Empfehlung an Ihren Arzt? Wenn er, ob er überhaupt solche Dinge ansprechen soll oder, wenn ja, wie? Haben Sie da irgendetwas, wo Sie sagen...*

*PAT: Ne, also ich muss, muss immer im, das kommt eigentlich auf den Patienten drauf an, ob er, ob er bereit ist darauf einzugehen. Also das ist von Fall zu Fall verschieden, da möchte ich eigentlich puuh kein, kein Urteil drüber abgeben, sagen wir es mal so. Er hat mich darauf angesprochen, ich bin darauf eingegangen. Beim nächsten Patient (xxx). Das liegt bei jedem Patienten selber.*

*INT: Würden Sie… hätten Sie eine Empfehlung an Ärzte, aufgrund Ihrer Erfahrungen, jetzt mit diesen Gesprächen?*

*PAT: Die sollen hellhörig werden, wenn die, wenn die Patienten irgendwie (--) ähm, dementsprechend Sachen von sich geben.*

*INT: Da soll ein Arzt drauf achten und dann drauf eingehen?*

*PAT: Ja, drauf achten genau, genau. Und wenn äh, eventuell, ich weiß jetzt nicht wie das ist, ob Probleme kommen oder irgendwie sowas, oder wenn er sich sehr schlecht fühlt, sowas irgendwie, dann darauf eingehen.*
